# Observational study of the relationship between volume and outcomes using data from the National Audit of Cardiac Rehabilitation

**DOI:** 10.1136/openhrt-2015-000304

**Published:** 2015-10-30

**Authors:** Patrick Doherty, Alexander S Harrison, Mike Knapton, Veronica Dale

**Affiliations:** 1 Department of Health Sciences, University of York, York, UK; 2 British Heart Foundation, London, UK

**Keywords:** CORONARY ARTERY DISEASE, QUALITY OF CARE AND OUTCOMES

## Abstract

**Objective:**

Cardiac rehabilitation (CR) is an evidence-based intervention delivered by a wide range of high-volume and low-volume centres; however, the extent of volume–outcome relationship is yet to be studied. There is a lack of consensus about the effect of volume on outcomes, with evidence of mixed effects in acute and chronic care. The aim of this study is, to investigate the extent of association of outcomes in CR with patient volume.

**Methods:**

Data was validated and extracted from the national audit from 2012 to 2013 for each CR centre. Volume was calculated as the total number of patients entering outpatient CR. Hierarchical multiple regression models were used to test for relationships between volume and outcomes. The outcomes included body mass index, blood pressure, psychosocial well-being, cholesterol, smoking cessation and physical activity. The analyses were adjusted for centre and patient characteristics and confounders.

**Results:**

The number of patients included in the volume analysis was 48 476, derived from 178 CR centres. The average age per centre was 66 years with a 70% male distribution of patients enrolled. Regression analysis revealed no volume–outcome relationship, additionally no statistical significance existed.

**Conclusions:**

Unlike cardiac surgery this study, after accounting for staffing, age, gender and comorbidity, shows no effect of volume on outcome following CR delivered by high-volume and low-volume programmes. Based on our data there is no support for centralisation of services. Our findings and methodology can be used as a benchmark for future volume–outcome relationship studies in CR.

**Key questions**

**What is already known about this subject?**

- Current literature supports a positive volume effect, with increasing patient throughput leading to improved outcomes in cardiac surgery but not in cardiac rehabilitation (CR).

**What does this study add?**

- This is the first volume–outcome relationship (VOR) study in the UK of CR. The methodology takes account of potential confounders such as age, gender, comorbidities and staff details.

**How might this impact on clinical practice?**

- Unlike the positive cardiac surgery VOR findings in favour of high volume, our study suggests that clinical strategies to optimise uptake to CR could be achieved through either approach.

## Introduction

Research from more than 45 clinical trials has shown that cardiac rehabilitation (CR) is a clinically effective secondary prevention programme leading to a significant reduction in premature cardiac mortality (26%; 95% CI 13% to 37%), total mortality (13%; 95% CI 1% to 25%) and improved quality of life.^1^ ^2^ CR is also a cost-effective therapy with an estimated cost per life year gained of less than £2000.^3^ National recommendations for CR and National Institute for Health and Care Excellence (NICE) guidelines state that programmes should be comprehensive including education, support with health behaviour change and exercise training and should be delivered by a multidisciplinary team (MDT).^2^ ^4^

The National Audit of Cardiac Rehabilitation (NACR), which is funded by the British Heart Foundation, collects clinical data from programmes allowing it to monitor and report on the quality of CR services in the UK. As with other health services; the size, resources and the extent of patient throughput varies across CR programmes. The extent of this variability, demonstrated in the literature and through the UK national audit, could give rise to a potential volume–outcome relationship (VOR) in CR.^5^ ^6^ With respect to volume expectations only the Scottish Intercollegiate Guidelines Network (SIGN 57) states a value for the delivery of MDT CR of 500 patients per year which is based on expert opinion rather than clinical outcome assessment.^7^

VOR have been investigated in other areas of cardiology and cardiac surgery identifying that large volume centres are associated with better outcomes.^8^ ^9^ This has led to significant centralisation of cardiac surgery services with specific staffing requirements and resources being made available for large volume centres only.^10^ The source of the relationship is believed to relate to higher volume of patients resulting in institutional experience, selective referral and improved process of care at higher volume institutions.^8^

It is expected that this volume effect may be mirrored in CR; this is because the quality of care may improve with increased patient throughput. There is an underlying assumption that ‘practice makes perfect’ which should mean that high-volume CR leads to a positive improvement in outcomes.^8^ There is a caveat to this in that national guidance and recent trial data from the UK express concern about the quality of CR delivery in routine practice.^4^ ^5^ ^10^ ^11^ There are national recommendations that CR is based on assessment and is delivered to a minimum standard by a MDT.^4^ ^10^ This team should implement risk factor management and facilitate health-related lifestyle changes in an increasingly multimorbid patient population. If a VOR were to be identified, then perhaps this finding would prompt policy for increased CR centralisation.

There is little VOR-specific research in CR. The one UK study relates to exercise class size rather than total volume and concludes that smaller class sizes was associated with increased mortality.^5^ In the context of risk factor outcomes one study from a similar care approach to CR (eg, psychiatric care) found a detrimental effect of volume on outcome, with increased hospital readmission in larger centres (OR of 30 day readmission, 3.0; 95% CI 2.8 to 3.2).^12^

Current CR although effective, has been shown to have low uptake with over half of eligible patients not receiving the programme.^10^ A possible method of improvement is to increase accessibility; this could be done through increasing number of centres.^13^ Although this study is not looking directly at accessibility, an offshoot is that changes to the way CR is run, large volume centres or many small volume centres, will impact accessibility.

Our study aims to investigate the relationship between volume of patients seen per year, with an experimental hypothesis that a positive VOR exists in CR.

## Methods

### Data collection

The analyses were conducted using individual patient data collected from the UK NACR 1 April 2012 to 31 March 2013. The NACR is a routinely administered audit within the National Health Service, which has approval to collect anonymised patient data for a range of clinical variables.^10^ The data is hosted by the Health and Social Care information Centre (HSCIC), to which approval is granted annually to use this data to monitor and report on the quality of CR. The audit collects data for patients who undergo CR in the UK including details of the patients initiating event, treatment, risk factors, medication, patient demographics and outcomes. UK CR is administered based on the British Association for Cardiovascular Prevention and Rehabilitation (BACPR) national guidelines which aim to reduce cardiovascular risk and promote quality of life through coordinated core components of cardiovascular disease prevention and rehabilitation using exercise training (moderate intensity twice weekly), diet and education support.^4^

Patients were included in the analyses, if they started CR and been assessed at baseline and had follow-up data at an assessment 2 (post-CR). This observational study was reported following the guidelines of the Strengthening the Reporting of Observational Studies in Epidemiology (STROBE).^14^

In addition to electronic data collection reported above, staffing details, per centre, were collected from the annual NACR paper survey, which collects data on types of staff, hours worked and numbers of staff per programme. The multidisciplinary nature of a CR programme was defined by having a minimum of three different professionals working in the team.

Nine key clinical outcome measures, deemed as important for risk factor management and routinely reported by the NACR were selected. These patient outcomes form part of national CR minimum standards.^4^ The outcomes were body mass index (BMI), blood pressure (BP), psychosocial health (Hospital Anxiety and Depression Scale (HADS) scores: anxiety and depression),^15^ total cholesterol, a measure of exercise capacity through the incremental shuttle walk test (ISWT), smoking status (yes/no) and self-reported moderate physical activity (PA) (150 min/week; yes/no) conforming to the Department of Health guidelines for 19–64 and 65+ age groups.^16^

Volume was defined as the total number of patients who had undergone baseline assessment and entered the standard core delivery of CR which in the UK is delivered as an outpatient service. This measure was used as it reflects the number of patients assessed (eg, starting CR) and the associated staffing requirements delivering the service.

### Statistical analysis

The analyses were conducted in STATA 13. The data was hierarchical with patients nested within centres, multilevel models were used for the analyses, with CR centre treated as a random effect. Volume, the number of patients with a baseline assessment per centre, was included as a continuous variable. A selection of known confounding factors reported in the literature was used as covariates in the analyses. These were age, gender, number of comorbidities and staffing details. The staffing details included total staff hours and whether the centre met the multidisciplinary criteria of three or more professional groups.^9^ ^12^ ^17^ Comorbidities are any of 19 commonly associated conditions that patients who undergo CR have, such as angina, diabetes and cancer. These comorbidities are routinely collected by the NACR and reported in the national statistical report.^6^ Owing to the high level of heterogeneity in the length of CR, duration could not be used as a possible confounder in this analysis, additionally the number of sessions is a new variable in the NACR since 2014. The models were also adjusted for the baseline value of the dependent variable in the model.

A linear mixed model, accounting for centre variation by hierarchical modelling, was used to assess the extent by which volume determined outcome for BMI, HADS Anxiety and Depression and a mixed model for categorical data was used for smoking and PA. Patients were included in the outcomes analysis, if they had complete data, that is, baseline characteristics recorded and had follow-up data at an assessment 2 (post-CR). Data model checking was performed to ensure that the models were a good fit, through assumptions associated with the regressions.

## Results

### Study population

**Figure 1:**
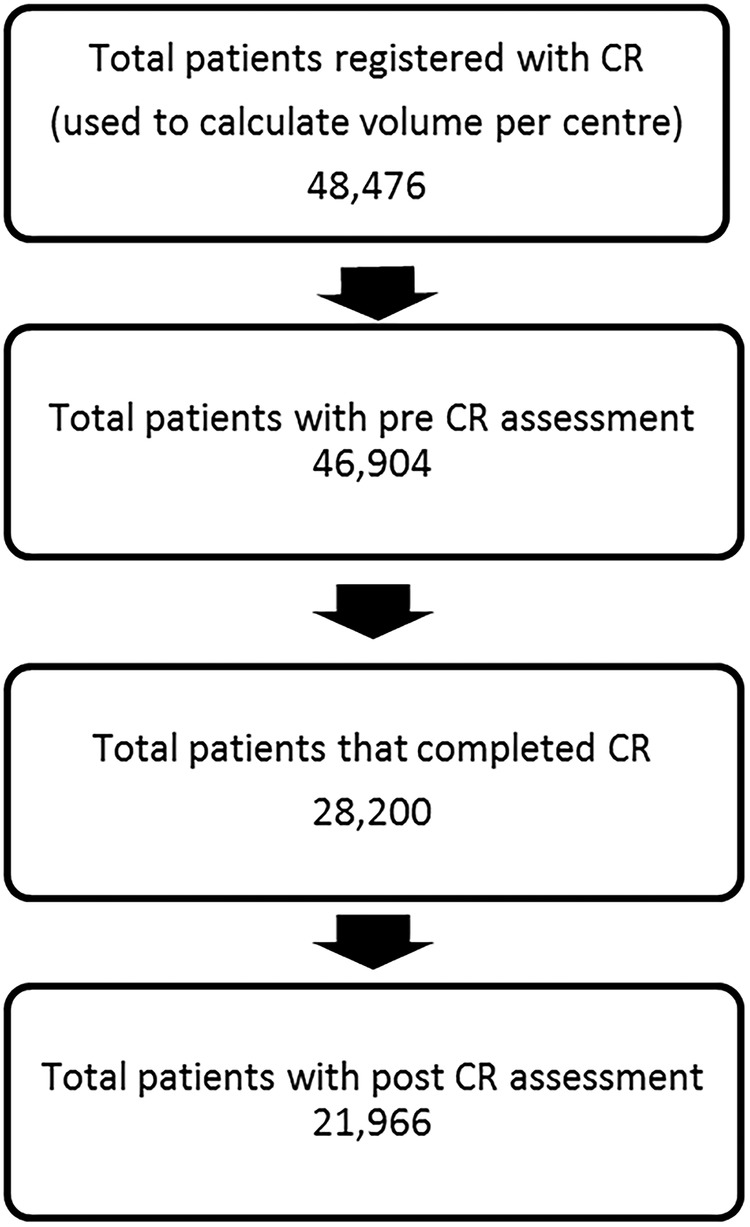
A flow diagram showing the total population in the data set with subsequent numbers for precardiac rehabilitation (CR) assessment, completed CR and patients with a follow-up assessment.

The study population is summarised in figure 1 based on a total of 48 476 patients included in the volume measure from 178 centres. The population was 70% male (34 067), with an average age of 66 years (SD=12.37) from postmyocardial infarction, coronary artery bypass grafting and percutaneous coronary intervention. Median comorbidity of the population was 1. Of the total included patients in the volume denominator, 21 966 also completed the full rehabilitation programme and attended post-CR assessment.

Baseline characteristics were collected for patients at the start of the programme (table 1) showing that the population was representative of patients with cardiovascular disease with above average baseline measures for BMI and correspondingly low levels of PA.

**Table 1:** Baseline characteristics for each of the nine clinical measures for the total CR population

Pre-CR assessment	Mean	SD	N
BMI (kg/m^2^) mean	28.12	(5.89)	25 385
Systolic BP (mm Hg) mean	128.92	(20.99)	32 273
Diastolic BP (mm Hg) mean	73.37	(12.17)	31 324
Total cholesterol (mmol/L) mean	4.65	(1.31)	19 491
HADS Anxiety score mean	6.8	(3.82)	22 135
HADS Depression score mean	5.8	(3.43)	20 329
ISWT (m) mean	338.36	(168.28)	5011
Percentage of patients smoking at baseline	14.65%		21 576
Percentage of patients achieving physical activity	12.1%		5878

BMI, body mass index; BP, blood pressure; CR, cardiac rehabilitation; HADS, Hospital Anxiety and Depression Scale; ISWT, incremental shuttle walk test.

Only patients with pre-CR and post-CR assessments were included in the analysis. Table 2 shows a comparison between pre and post assessment across all measures. The exercise capacity measure (ISWT metres) is the only measure that has a reported minimum clinically important difference measure, which is above 70 m in CR populations.^18^ As shown in table 2 on average the population is achieving above this figure by 24 m.

**Table 2:** Baseline and outcome values for patients with valid follow-up included in the analysis

	Pre	Post	
	Mean	Mean	N
BMI (kg/m^2^) mean	28.01	28.02	11 332
Systolic BP (mm Hg) mean	128.92	128.99	11 864
Diastolic BP (mm Hg) mean	74.15	73.70	11 843
Total cholesterol (mmol/L) mean	4.69	4.01	5003
HADS Anxiety score mean	7.08	6.33	8872
HADS Depression score mean	6.09	5.37	7207
ISWT (m) mean	350.52	444.26	2560
Percentage of patients smoking at baseline	5.4%	4.3%	1874
Percentage of patients achieving physical activity	36.9%	75.5%	2164

BMI, body mass index; BP, blood pressure; HADS, Hospital Anxiety and Depression Scale; ISWT, incremental shuttle walk test.

**Figure 2:**
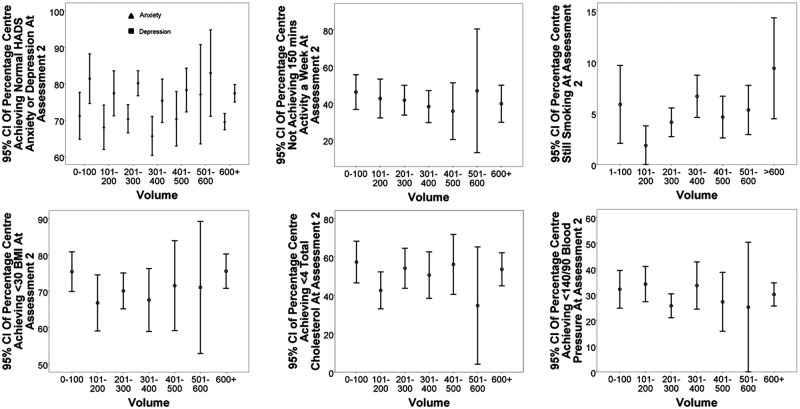
The volume per centre plotted against clinical outcomes which included Hospital Anxiety and Depression Scale (HADS) score, exercise 150 min, smoking, body mass index (BMI), blood pressure and total cholesterol. The measure of outcome is the percentage of participants reaching target boundaries. The error bars represent the 95% CI per volume category.

To account for the variation and staffing between centres in the analysis, total staffing hours and whether the centre met the three MDT professional minimum criteria were included as covariates. Overall 77.7% of centres met the minimum criteria, and the average number of total staff hours per week was 197.4 (SD=116.03), which equates to approximately 5.5 full time staff members. The average number of patients in a centre was 368 patients with a large SD (196.92) leading to a range from a minimum of 42 in the smallest centre to 1417 in the largest centre. The median number of patients was 341. Figure 2 shows the volume per centre plotted against of nine outcomes, such as HADS Anxiety and Depression, BMI and cholesterol. The measure of outcome is the percentage of patients who reached the target reading for each outcome per centre. The error bars represent the 95% CI based on the collection of different centres inside each group of volumes, 1–100 and 101–200. The target reading was a measure of the scale for the continuous outcomes (HADS<8, BMI<30 kg/m^2^, cholesterol <4 mmol/L, BP<140/<90 mm Hg), whereas the dichotomous variables, smoking and PA, were ‘not smoking’ and reaching 150 min exercise per week, respectively. The target readings were created using the CR outcomes used in the NACR annual report.^6^ This could not be performed for the ISWT as yet there is no suggested minimum target for patients at baseline. In all the clinical outcomes shown in figure 1, there was no clear association between volume and the extent of patient outcomes.

The results from the regression analysis are in table 3. For the predictor variable, total volume per centre, there were no significant relationships observed in any of the outcomes.

**Table 3:** Regression coefficients and OR for volume from the mixed model regression of the nine clinical outcomes

	Volume coefficient×10^−3^	Significance*	95% CI×10^−3^
BMI	0.1	0.820	–0.65 to 0.515
Systolic BP	0.242	0.929	–5.12 to 5.61
Diastolic BP	1.88	0.191	–0.94 to 4.69
Cholesterol	0.223	0.375	–0.27 to 0.715
HADS Anxiety	0.027	0.933	–0.66 to 0.609
HADS Depression	0.272	0.393	–0.35 to 0.895
ISWT (m)	−0.037	0.514	–1.15 to 0.075

	**OR**	**Significance**	**95% CI**

Smoking	1.001	0.276	0.998 to 1.002
Physical activity	1.001	0.994	0.999 to 1.001

*Analyses adjusted for age, gender, comorbidities, staffing profile and baseline measurement.

BMI, body mass index; BP, blood pressure; HADS, Hospital Anxiety and Depression Scale; ISWT, incremental shuttle walk test.

Model checking was performed to ensure that the models were a good fit for the data. The covariates included such as age, gender and comorbidities made no notable difference to the outcomes with volume having no significant relationship. The analysis was run with and without staff level details, this led to no change in the VOR results.

## Discussion

The principal finding of this study was that, based on clinical outcomes from the NACR data there is no evidence to support a VOR in routine CR. The study used hierarchical regression to investigate whether volume influences a range of patient outcomes reported by CR. No statistically significant associations were found for VOR. Model checking analysis showed the model was a good fit requiring no significant changes to the results.

In this study's population there were 48 476 patients who met the volume definition used for this study through starting outpatient CR. The participating population had a similar distribution of males to females as the most recent Cochrane review of clinical trials in CR with around 30% females. The age of patients was on average 10 years older in routine practice compared with the Cochrane review.^1^ Our analysis accounted for the potential for age to impact on the outcome.

Our analysis which aimed to investigate volume and outcome is best interpreted in the context of the BACPR minimum standards which state that CR should be delivered by a MDT.^4^ The staff details inputted in the analysis included the total number of hours worked and whether the centre had three or more MDT professionals. Despite having National and European guidelines defining the service specification for CR this study showed that the staffing hours and the MDT profile varies substantially.^4^ ^12^ ^19^

Much of the literature concerning volume, such as the work by Gammie *et al*^8^ and Seperhripour and Athanasiou^9^ in cardiac surgery showed a positive VOR. The only UK-specific study that included an aspect of volume (eg, exercise class sizes) found a non-significant but positive trend in favour of increased mortality with smaller volume.^5^ The research undertaken by Lee and Lin^12^ in Japan adds complexity to any conclusion by showing that tailored patient care (eg, low volume), such as that seen in psychiatric services, are beneficial at reducing hospital readmissions.

The evidence from our national study of routine CR, which accounted for known confounders, is unable to support either a negative or positive VOR. This study accounted for five well-understood covariates, known to influence cardiovascular outcomes, which boosts confidence in our findings but we cannot rule out interaction by other potential confounders such as patient case severity and the skills, experience and training of MDT staff.

One of the drivers for this research was that with increasing emphasis from policymakers to maximise throughput and efficiency, the volume within centres may need to increase. In many areas of cardiology a change has already been made to increase the number of high-volume centres. This has been proposed for CR as high volume is considered good practice.^10^ There may be other benefits to increasing the number of high-volume centres such as reduced costs and improved patient access; however, improved clinical outcome is not supported as a high volume benefit in CR in the UK.

Clark *et al* argue that there is a severe problem with current CR programmes, which is poor accessibility. The article discusses how accessibility maximised through more accessible programmes would improve uptake to CR. The conclusion to their work and how exactly accessibility could be maximised is yet to be studied.^13^ Currently only 44% of eligible patients receive CR meaning that over half of all patients are not taking up evidence-based CR.^10^ Our findings suggests that size does not matter and smaller throughput programmes offer similar outcomes to larger ones. There may be unforeseen negative consequences, in terms of accessibility, when centralising programmes to improve productivity and clinical outcomes. Future research is required to evaluate innovation in clinical practice around a localised and centralised solutions aimed at increasing accessibility and outcomes.

### Limitations

In contrast to the recommended national minimum standards there was significant under-reporting of the clinical outcomes. Standard 4 of BACPR standards, states that all patients undergoing CR should have ‘Reassessment carried out upon completion of the CR programme to determine achievements of goals’. Based on NACR data, of all patients who completed CR, 32% did not have a post-CR assessment recorded. This shortfall will become less of an issue going forward as the BACPR and NACR have initiated a national certification scheme which has mandated post-CR assessment as a clinical standard.

Finally, the study used postrehabilitation assessment, after a median duration of 8 weeks of intervention, which albeit meets the minimum standards it may be insufficient time for certain risk factors to change.^17^ ^19^

## Conclusion

This study aimed to investigate whether there was an association between the volume of patients starting CR at a centre and clinical outcome.

Contrary to the literature this analysis showed no evidence to support any direction of a VOR within current UK CR.

This research has developed a robust approach to audit-based research and established a UK baseline from which future longitudinal audit-based research can be conducted. Future NACR research, involving data linkage with cardiology registries, aims to investigate the interaction between patient case severity and outcome in those attending and not attending CR.
